# A Radio Environment Maps Estimation Algorithm based on the Pixel Regression Framework for Underlay Cognitive Radio Networks Using Incomplete Training Data

**DOI:** 10.3390/s20082245

**Published:** 2020-04-15

**Authors:** Xu Han, Lei Xue, Ying Xu, Zunyang Liu

**Affiliations:** Electronic Countermeasure College, National University of Defense Technology, Shushan District, Hefei 230037, China; hanxu17@nudt.edu.cn (X.H.);

**Keywords:** wireless communication, deep learning, cognitive radio networks, generative adversarial networks, image reconstruction

## Abstract

In the underlay cognitive radio networks, the radio environment maps (REMs) estimation is the main challenge in sensing the idle wireless spectrum resources. Traditional deep learning-based algorithms estimate the REMs on the basis of the high-quality, large-scale complete training images. However, collecting the complete radio environment images is time-consuming and requires a numerous number of power spectrum sensing nodes. For this reason, we propose a generative adversarial networks-based pixel regression framework (PRF) for underlay cognitive radio networks. The PRF algorithm relaxes the requirement of the complete training images, and estimates the radio environment maps only on the basis of the incomplete REMs images, which are easier to be collected. First, we transform the radio environment maps estimation task into a pixel regression task through the color mapping progress. Then, to extract helpful information from the incomplete training data, we design a feature enhancing module for the PRF algorithm, which intelligently learns and emphasizes the important features from the training images. Finally, we use the trained pixel regression framework to reconstruct the radio environment maps in the target area. The proposed algorithm learns accurate radio environment characteristics from the incomplete training data rather than making direct biased or imprecise radio propagation assumptions as in the traditional methods. Thus, the PRF algorithm has a better REMs reconstruction performance than the traditional methods, as verified by simulations.

## 1. Introduction

Cognitive radio network (CRN) is a promising technology to optimize the utilization of the spectrum resources [1]. In the cognitive radio network, unlicensed users can access the spectrum holes in time, frequency and space or any of their combinations, provided they cause no interference [2]. The users of the CRNs change their transmitting parameters intelligently to solve the spectrum scarcity problem [3,4].

There are two types of users in the cognitive radio network: PUs (primary users) and SUs (secondary users) [5]. The primary users legally have the right to use the spectrum resources initially. As for the secondary users, they try to access the PUs’ working band dynamically by intelligently sensing and utilizing the unused licensed band.

An important radio resources allocation scheme is the underlay cognitive radio network, which is also named as the spatial reuse CRN [6,7]. In the underlay cognitive radio network, the SUs can safely use the licensed band if the interference does not exceed the thresholds of the primary users because of the power attenuation in the wireless radio propagation path [8]. The above radio resources allocation scheme improves the spectrum efficiency by utilizing the wireless “white space”, which is about the idle licensed spectrum resource in the space and frequency domains. The underlay CRN has strict requirements of the working power of the SUs, which can significantly improve the utilization of the radio resources based on the efficient and intelligent spectrum management.

In the underlay cognitive radio network, we adopt the radio environment maps (REMs) to display the primary users’ power spectrum (PS) in the CRN area. It is a visible map of the wireless environment, which adds the PS information into the spatial map. Based on the estimation of the REMs, we can handle the conflict between the SUs and PUs and maximize the utilization of the spectrum resources in a particular CRN region.

Estimating and utilizing the radio environment maps is extraordinarily helpful in wide-area CRN [9]. To reduce the interference to PUs, the SUs intelligently change their transmitting power on the basis of the REMs, allowing the remote users to dynamically utilize the idle radio resources [10,11].

**Figure 1:**
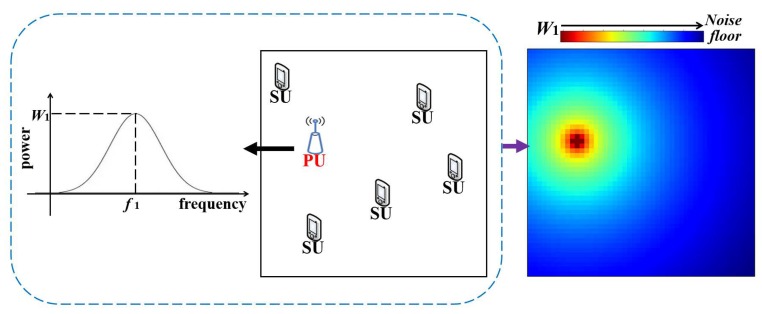
From left to right: The power spectrum of the PU; the distribution of the receiving SUs and the transmitting PU; the radio environment map of the target area in frequency $f_{1}$.

As shown in Figure 1, for estimating the radio environment maps, the general setting includes several transmitting PUs and receiving SUs. We suppose that they are uniformly distributed in the target area. In the AWGN (additive white Gaussian noise) environment, several secondary users estimate the REMs in the target area collaboratively. The variance of the AWGN is assumed to be known, i.e., the noise floor in Figure 1. In addition, we assume that the receiving PS of SUs, their locations are known. However, the information of the PUs, i.e., their locations and their working power spectrum, is supposed to be unknown. Our goal is to estimate the above unknown parameters on the basis of the SUs’ parameters, i.e., the radio environment maps in the target area.

In fact, the above goal is undetermined. There are infinite REMs which can satisfy the above known SUs’ parameters. To reduce the solution space, we should utilize the prior information of the radio environment in the target region. However, traditional methods [12,13,14,15] usually make direct imprecise or biased radio propagation assumptions about the radio environment, which lead to the inaccurate PSD maps estimation results.

**Figure 2:**
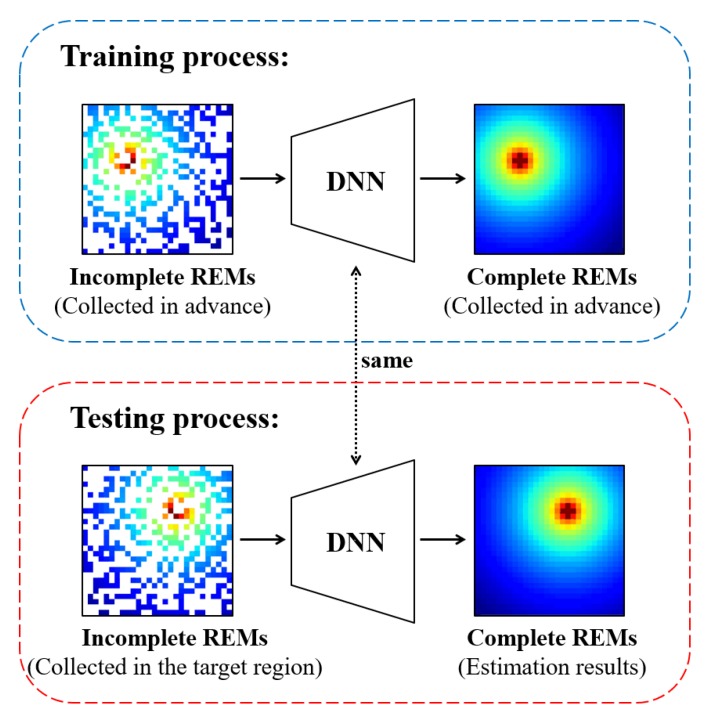
The sketch map of the MEGANs algorithm. The deep neural network (DNN) is trained to learn from the paired training data, i.e., the incomplete REMs and the complete REMs, to estimate the radio environment maps.

As a significant method of the artificial intelligence (AI), deep learning technology has irreplaceable advantages in the radio environment maps estimation task [16]. By exploiting the latent propagation features of the wireless environment, we can get the prior knowledge on the basis of the deep neural network (DNN) through supervised training (Figure 2), which makes the radio environment maps estimation results more intelligent and precise. However, the studies on deep learning-based REMs estimation algorithms are just in primary stage and deserve more research.

In our previous work [16], we proposed a deep learning-based maps estimation generative adversarial networks (MEGANs) to estimate the radio environment maps. By learning and utilizing accurate radio propagation features from the complete training data, the proposed MEGANs algorithm provides a more accurate estimation performance than the conventional methods.

As shown in Figure 2, the paired training data, i.e., the incomplete REMs and the complete REMs, are the key factors to train the deep neural network (DNN). The training data must be independent and identically distributed (i.i.d.) as the REMs in the target area. Thus, we have to collect the power spectrum in every position of the target region in advance. However, collecting the complete REMs is not an easy task, which is time-consuming and requires a numerous number of power spectrum sensing devices. The hard-collected training data have become one of the most challenging obstacles for the development of the deep learning-based REMs estimation algorithms.

Compared with the complete REMs, the incomplete REMs are easier to be collected. We can simply collect them in advance by setting a small amount of power spectrum sensing devices in the target region. Training the deep neural network to learn from the incomplete REMs training data is a more realistic way, which has not been reported until now.

In this paper, we propose a generative adversarial network (GAN) -based REMs estimation algorithm named pixel regression framework (PRF) for underlay CRNs. Compared with our previously proposed MEGANs [16], the PRF algorithm relaxes the requirement of the complete training images, and can estimate the radio environment maps only on the basis of the incomplete REMs training data, which are much more realistic to be collected. Compared with MEGANs, the original contributions are shown as follows:

- To estimate the REMs for underlay CRNs, we propose a GAN-based PRF algorithm using only incomplete REMs training data. Using the incomplete training data to estimate the radio environment maps in the target region has not been reported until now.
- In the MEGANs algorithm, the discriminator is trained to distinguish if the input complete images are from the real complete training data set or from the REMs estimated by the generator. However, unlike the MEGANs settings, we only have a dataset of incomplete REMs training images. In this paper, we propose a pixel regression framework in which the discriminator is trained to identify if the input incomplete images are sampled from the real complete REMs or from the REMs estimated by the generator.
- Compared with the complete training data, the incomplete REMs images contain relatively less information. To improve the feature extraction ability of the neural network in MEGANs, we design a feature enhancing module in our PRF algorithm. The proposed module can extract more information from the incomplete training data and intelligently emphasize the important features of the radio environment maps.

The rest of our paper is organized as follows. In Section 2, we introduce related works of the REMs estimation task. We build the REMs model in Section 3. We propose a GAN-based pixel regression framework for underlay CRNs in Section 4. In Section 5, we conduct several simulations to test the PRF algorithm. In Section 6, the research findings are concluded.

The acronyms in this paper are shown in Table 1.

## 2. Related Works

The radio environment map is a powerful tool to determine the PUs’ signals across a finite geographical area. On the basis of the REMs, we can obtain the distribution of the signal strength in the target region. As for the applications of the radio environment maps, DARPA developed its advanced radio frequency mapping program, i.e., Radio Map, to achieve the real-time sensing of the spectrum resources across space and frequency domains [17]. Based on a joint tensor completion algorithm, the authors in [18] build a database to get the idle radio resources information. In [19] exploits the unused spectrum and determines the maximum permitted transmitting power on the basis of the radio environment maps. In addition, high time resolution spectrum occupancy measurements and analysis are presented in [2] for 2.4 GHz WLAN signals. The authors propose a custom-designed wideband sensing engine to record the received power of signals, and its performance is presented to select the decision threshold required to define the channel state (busy/idle).

To solve the undetermined REMs estimation problem, initial efforts have been made by utilizing the prior information or assumptions of the radio environment in the target area. Conventional radio environment maps estimation methods include spatial interpolation methods [12,13,14,15,20] and the deep learning-based methods [16].

The spatial interpolation methods include the inverse distance weighted (IDW) interpolation algorithm and the Kriging spatial interpolation algorithm. The inverse distance weighted interpolation supposes that the PS only depends on the distance $d_{idw}$ between the receiving SUs and the interpolation locations [14,15]. The power value of the inverse distance is ${\left(\frac{1}{d_{idw}}\right)}^{p_{id}}$. $p_{id}$ is a pre-selected constant, which controls the weights of the receiving SUs upon other locations. In fact, the IDW method is not related to any real physical fact. It is hard to decide whether a certain $p_{id}$ is proper or not.

As for the Kriging interpolation method, it estimates the radio environment maps with the weighted additions of the known PS parameters on the basis of the semi-variogram functions. We can regard the above functions as the latent features of the wireless environment in the target area [12,13], which measures the relationships between the average power value differences of different nodes and the distances separating them [20].

All the above algorithms perform well in some simple environments. However, the real wireless environment is quite complicated. In the practical radio environment, the signal is attenuated in a random fashion. The attenuation is mainly caused by the radio propagation loss, the shadow fading, the multi-path effect, etc. Under the superposition of the above attenuation styles, building a high-accuracy mathematical model for the radio environment is quite difficult. However, all the above traditional REMs estimation methods make direct assumption about the radio environment. Biased or inappropriate hypotheses will lead to inaccurate radio environment maps estimation results. For example, the IDW interpolation algorithm assumes that the power spectrum only depends on the distance between the PUs and SUs [14,15]. The IDW method has a poor REMs estimation performance in the urban area, where there is severe shadow fading effect.

Deep learning is a promising technology to estimate the radio environment maps. On the basis of the deep neural networks, we can obtain the prior knowledge of the wireless environment by extracting the latent propagation features from the training data set, which makes the REMs estimation results more intelligent and precise. However, there are few studies which focus on deep learning-based REMs estimation algorithms [16].

In our previous work [16], we proposed a deep learning-based maps estimation generative adversarial networks (MEGANs) to estimate the radio environment maps. Generative adversarial networks (GANs) are recently introduced as a powerful framework to handle regression problems in deep learning [21]. There are two components in the generative adversarial networks: the generator (*G*) and the discriminator (*D*) [22]. The strategy of MEGANs is defining a game between the generator and the discriminator [16]. The generator is trained to generate a high-accuracy estimation of the REMs and fool the discriminator; the discriminator is trained to decide if the generated REMs are true or false. By learning and utilizing accurate radio propagation features from the complete training data, the proposed MEGANs algorithm provides a more accurate estimation performance than the conventional methods.

In the MEGANs, the generator and the discriminator are trained on the basis of the complete training data. However, collecting the complete REMs is time-consuming and requires a numerous number of sensing nodes. In this paper, we propose a novel GAN-based REMs estimation algorithm named PRF algorithm. The proposed method can extract helpful information from the incomplete REMs training data, which are relatively easy to be collected.

## 3. Radio Environment Maps Model

We assume that there are $N_{P}$ transmitting PUs and $N_{S}$ receiving SUs. They are uniformly distributed in a square target region ***T***. The SUs and PUs are located at ${\left\{(x_{i},y_{i})\right\}}_{i=1}^{N_{S}}$ and ${\left\{(p_{i},q_{i})\right\}}_{i=1}^{N_{P}}$ respectively. The receiving secondary users try to estimate the radio environment maps of the target area under the AWGN with a known variance $\sigma^{2}$. We use ${\left\{{\;\;\;\Phi \;\;\;}_{i}\left(f\right)\right\}}_{i=1}^{N_{S}}$ to represent the receiving power spectrum of the secondary users. ${\left\{\Psi_{i}\left(f\right)\right\}}_{i=1}^{N_{P}}$ denotes the transmitting power spectrum of the primary users.

We use $P_{S}\left(f;x,y\right)$ to denote the power spectrum at location (x,y). $l_{\left(p,q)\to (x,y\right)}$ denotes the unknown radio attenuation function from the PU’s location (p,q) to (x,y). As shown in Equation (1), we adopt the same REMs regression model used in our previous work [16].

(1) $$\begin{array}{c} \begin{array}{cc} \; & P_{S}\left(f;x,y\right)=\sum_{i=1}^{N_{P}}l_{\left(p_{i},q_{i}\right)\to \left(x,y\right)}\Psi_{i}\left(f\right)+\sigma^{2}\;,\forall \left(x,y\right)\in T\\ s.t.\; & \Phi_{j}\left(f\right)=\sum_{i=1}^{N_{P}}l_{\left(p_{i},q_{i}\right)\to \left(x_{j},y_{j}\right)}\Psi_{i}\left(f\right)+\sigma^{2}\;,j=1,2,\ldots ,N_{S} \end{array} \end{array}$$

We suppose that $N_{S}$, ${\left\{(x_{i},y_{i})\right\}}_{i=1}^{N_{S}}$, ${\left\{{\;\;\;\Phi \;\;\;}_{i}\left(f\right)\right\}}_{i=1}^{N_{S}}$ are known, but $N_{P}$, ${\left\{(p_{i},q_{i})\right\}}_{i=1}^{N_{P}}$, ${\left\{\Psi_{i}\left(f\right)\right\}}_{i=1}^{N_{P}}$ are unknown. Our task is to estimate the above REMs model on the basis of the known parameters.

In fact, the above goal is undetermined. There are infinite REMs which can satisfy the constraints in Equation (1). To compress the REMs solution space, we propose a pixel regression framework to extract helpful knowledge of the wireless environment from the incomplete REMs training data. Then, we utilize the extracted information as the prior knowledge to estimate the radio environment maps.

## 4. The PRF-Based REMs Estimation Algorithm

### 4.1. Color Mapping

**Figure 3:**
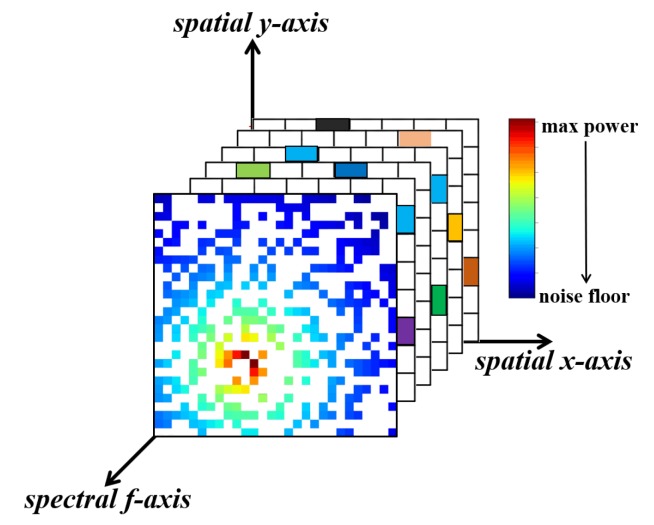
The incomplete, colored radio environment maps.

In the color mapping process, we divide the target region into N×N grids and assume that there is at most one user (one PU or one SU) in each grid. Then we normalize the secondary users’ receiving power spectrum, and map the power components of different frequencies to different colors uniformly, as shown in Figure 3. The white squares in the REMs are the grids where there are no secondary users. Our goal is to estimate the power values at the white squares.

On the basis of the color mapping process, we transform the REMs estimation task into a pixel regression task. Then, the efficient regression method—generative adversarial networks can be used to solve the REMs estimation problem.

### 4.2. The Pixel Regression Framework

In our previous work [16], we proposed a GANs-based algorithm named MEGANs to estimate the REMs in the target region. By learning from the complete training data set, the MEGANs algorithm achieves good REMs estimation performance. However, collecting the complete training data is not an easy task, which is time-consuming and requires a numerous number of power spectrum sensing nodes. Learning from the incomplete REMs training data to estimate the radio environment maps is a more realistic way.

**Figure 4:**
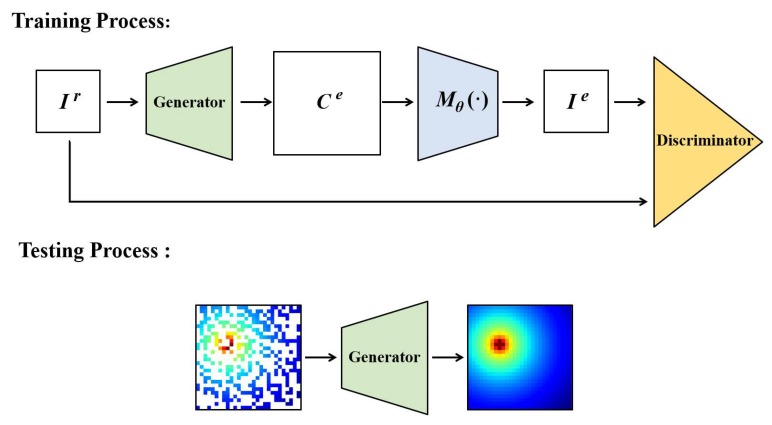
The pixel regression framework.

On the basis of the MEGANs, we propose an improved GANs-based algorithm named pixel regression framework, as shown in Figure 4. The proposed algorithm relaxes the requirement of the complete training images in MEGANs, and can extract useful information from the incomplete REMs training data.

Throughout, the superscript "*r*" denotes the true or real power spectrum distribution in the target area. Superscript "*e*" denotes the estimated or generated PS distribution from the generator. We use "*C*" to denote complete REMs images and "*I*" for incomplete REMs images. For example, $I^{r}$ denotes the true incomplete REMs images. In addition, we use $p_{c}^{r}$ to denote the underlying distribution of the true complete radio environment maps images, i.e., $C^{r}∼p_{c}^{r}$. Similarly, we use $p_{c}^{e}$ to denote the latent distribution of the estimated complete radio environment maps, i.e., $C^{e}∼p_{c}^{e}$.

On the basis of the generative adversarial network [22], the proposed PRF algorithm includes a generator (*G*), a discriminator (*D*) and a sampler ($M_{\theta }$), as shown in Figure 4.

In the PRF algorithm, we train *G* to learn the wireless radio propagation features from the training data, and produce accurate REMs estimation results, i.e., $G\left(I^{r}\right)=C^{e}$. As for the sampler $M_{\theta }$, it produces incomplete samples from the estimation results, i.e., $M_{\theta }\left(C^{e}\right)=I^{e}$. Regarding the discriminator, it is trained to identify if the input incomplete images are sampled from the real complete REMs or from the REMs estimated by the generator.

The strategy of the PRF algorithm is defining an adversarial game between *D* and *G* [22]. During the training process of the PRF algorithm, the identification ability of *D* and the estimation ability of *G* are continually improved until reaching a balance, where $p_{c}^{e}$ is an extremely close match to $p_{c}^{r}$, i.e., *D* can not identify if $I^{e}$ is sampled from $p_{c}^{e}$ or from $p_{c}^{r}$.

To achieve the above training plan, we adopt the objective function as follows:

(2) $$\begin{array}{cc} \min_{G}\max_{D}\;\; & E\left[D(I^{r})\right]\\ - & E\left[D(M_{\theta }\left(G\left(I^{r}\right)\right))\right]\\ - & \beta \cdot E\left[{\left({∥\nabla_{I^{l}}D\left(I^{l}\right)∥}_{2}-1\right)}^{2}\right]\; \end{array}$$

The third term is the gradient penalty in WGAN-GP [23]. It improves the training stability of the PRF algorithm. The coefficient of the gradient penalty is β. $I^{l}$ is the random linear interpolation of $I^{r}$ and $I^{e}$.

As mentioned above, the sampler $M_{\theta }$ is an important component in the proposed pixel regression framework, which produces incomplete samples from the estimation results. We should design $M_{\theta }$ according to the distribution of the power spectrum sensing nodes and the geographical environment of the target area ***T***. We list some examples of $M_{\theta }$ as follows.

- Random pixels sampler: Each pixel in $C^{e}$ is independently and randomly set to 0 with the probability θ. θ is uniformly distributed, i.e., $p_{\theta }∼U\left(\alpha_{0},1\right)$. $\alpha_{0}$ should be less than or equal to the proportion of the white squares in the real incomplete REMs images. For example, if the sensing nodes are uniformly distributed in the target area, the random pixels sampler should be adopted.
- Random pixels sampler with blank patches: On the basis of the above random pixels sampler, we set several patches of the input image to 0. The sizes of different patches are set according to the geographical environment in the target area. For example, if there are some buildings in the target area, and the sensing nodes are uniformly distributed outside the buildings, we should use the random pixels sampler with blank patches. The buildings in the target area are represented by the blank patches.

It should be noted that we do not use the same sampling locations as the true incomplete REMs images, i.e., the color grids’ locations of $I^{r}$ and $I^{e}$ are different in the training process of the PRF algorithm. The reasons are as follows:

(1) In our proposed algorithm, the discriminator is trained to identify if the input incomplete images are sampled from the real complete REMs $C^{r}$ or from the estimated REMs $C^{e}$. When we collect the true incomplete REMs for the training data set, the sensing nodes are set in a random way in the target area, i.e., the $I^{r}$ is an incomplete image randomly sampled from $C^{r}$. Thus, setting grids in $C^{e}$ to zero randomly is enough for training the discriminator. We do not have to force $I^{e}$ to have the same color grids’ locations as $I^{r}$.
(2) If we force $I^{e}$ to have the same color grids’ locations as $I^{r}$, the discriminator may be trained to identify if $I^{e}$ is equal to $I^{r}$ or not. Under the above conditions, the generator will generate the right values in the grids where there are sensing nodes, and will not pay attention to estimate the values in the grids where there are no sensing nodes. We take an extreme case as an example. If $I^{e}$ has the same color grids’ locations as $I^{r}$, the generator may tend to generate an image, which is identical to its input image, i.e. $G\left(I^{r}\right)=I^{r}$. Then, the generator can successfully fool the discriminator because $M_{\theta }\left(G\left(I^{r}\right)\right)=M_{\theta }\left(I^{r}\right)=I^{r}$. Thus, we do not use the same sampling locations as the true incomplete REMs images for the subsequent comparison in the discriminator.

### 4.3. The Structure of the Deep Neural Network

**Figure 5:**
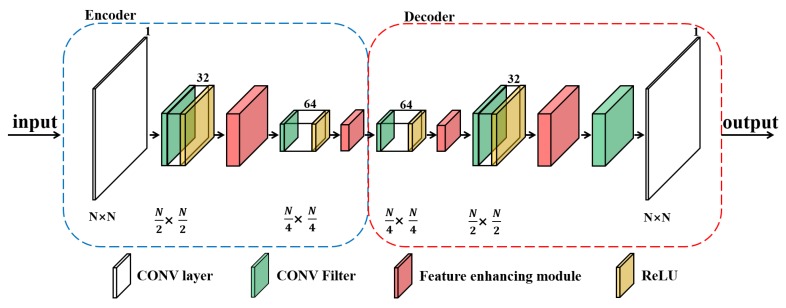
The neural structure of the generator in the pixel regression framework.

**Figure 6:**
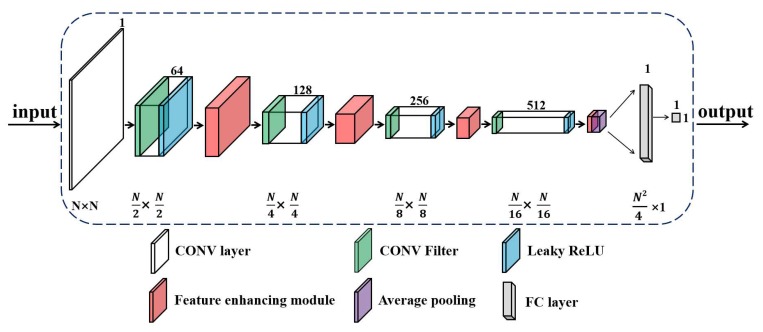
The structure of the discriminator in the pixel regression framework.

On the basis of the MEGANs [16], the improved neural structures of the generator and the discriminator in PRF algorithm are shown in Figure 5 and Figure 6.

Regarding the generator, the proposed structure is designed in the light of the auto-encoders [24]. The generator is trained to regress for the blank grids in the incomplete REMs images. The auto-encoders-based structure exploits the training REMs images and learns the latent wireless environment characteristics of the target area ***T***.

As for the discriminator of PRF, a deep convolutional neural structure is utilized to distinguish the true incomplete REMs from the estimated incomplete REMs (Figure 6). In addition, the discriminator helps the generator to strengthen the REMs images reconstruction ability. The convolution process in the discriminator exploits the latent features of the REMs images and enhances the identification performance to the estimated radio environment maps.

In MEGANs, the deep neural network is trained to extract useful information from the complete training images. However, compared with the complete training data, the incomplete REMs images contain relatively less information. To improve the feature extraction ability of the neural networks in MEGANs, we design a feature enhancing module in our PRF algorithm, as indicated by the red modules in Figure 5 and Figure 6. The proposed module can extract more information from the incomplete training data and intelligently emphasize the important features of the radio environment maps.

### 4.4. The Feature Enhancing Module in the Pixel Regression Framework

To extract the latent radio environment characteristics from the incomplete training images, we need to improve the feature extraction ability of the neural network. Adding more layers to the original neural network is a general method [21], but deeper neural network also brings difficulties to the training process [25].

**Figure 7:**
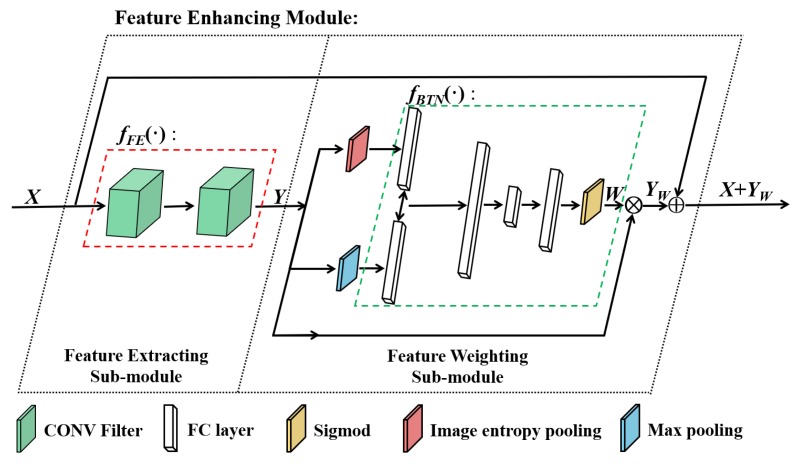
The structure of the feature enhancing module.

On the basis of the squeeze-and-excitation block [26], we design the feature enhancing module in the proposed PRF to enhance the approximation ability and the feature extracting ability of the deep neural network. The feature enhancing module includes two sub-modules: the feature extracting (FE) sub-module and the feature weighting (FW) sub-module. The details of the feature enhancing module are shown in Figure 7.

Regarding the FE sub-module, it only includes two convolutional layers, which deepen the neural network and extract more information from the incomplete training images. It should be noted that the two convolutional layers employ 3×3 kernel size with 1 padding, which maintain the dimensions of the inputs and outputs of the sub-module. The dimension maintaining process makes the proposed module a more generic extension, which can be added to the original neural network.

We assume that the input of the FE sub-module is $X\in R^{H\times W\times C}$. The function of the FE sub-module is $f_{FE}\left(\cdot \right)$. Its output is $Y\in R^{H\times W\times C}$, i.e.,$Y=f_{FE}\left(X\right)$.

As for the FW sub-module, it weights and emphasizes the important features and the detail features. The FW sub-module includes 3 main processes: the image entropy pooling, the max pooling and the fully connected bottleneck layers.

Regarding the image entropy pooling, we compute the channel-wise image entropy of the input image, which measures the information of different channels. The image entropy pooling process extracts the detail features of different feature maps. The fully connected bottleneck layers are trained to emphasize the feature maps on the basis of the above pooling results.

We use $e_{i}\in R$ to denote the *i* th channel’s image entropy of *Y*. We assume that the image entropy pooling function is $f_{IEP}\left(\cdot \right)$. The output of the pooling is $E\in R^{C\times 1}$. The image entropy pooling process is shown in Equation (3).

(3) $$E=f_{IEP}\left(Y\right)={\left(e_{1},e_{2},\ldots ,e_{C}\right)}^{T}$$

As for the max pooling process, we compute the channel-wise maximum value of the input image, which measures the prominent information of different channels. The max pooling process extracts the importance of different feature maps. The fully connected bottleneck layers are trained to emphasize the feature maps on the basis of the pooling results.

We use $m_{i}\in R$ to denote the *i* th channel’s maximum value of *Y*. The max pooling function is $f_{MXP}\left(\cdot \right)$. The output of the pooling is $M\in R^{C\times 1}$. The max pooling process is shown in Equation (4).

(4) $$M=f_{MXP}\left(Y\right)={\left(m_{1},m_{2},\ldots ,m_{C}\right)}^{T}$$

Regarding the fully connected bottleneck layers, they set different feature maps with different channel weights, which can be regarded as a self-attention function towards different channels.

The feature weighting process is shown in Equation (5) and Equation (6). We use $y_{i}\in R^{H\times W}$ to denote the *i* th channel of *Y* and its weight is $w_{i}\in R$. In addition, the bottleneck function is $f_{BTN}\left(\cdot \right)$ and its output is $W\in R^{C\times 1}$. We use $Y_{W}$ to denote the weighted results, and ⊗ denotes the channel-wise multiplication.

(5) $$W=f_{BTN}\left(E,M\right)=\left(w_{1},w_{2},\ldots ,w_{C}\right)$$

(6) $$Y_{W}=Y⊗W=\left(w_{1}y_{1},w_{2}y_{2},\ldots ,w_{C}y_{C}\right)$$

In order to deepen the neural network and avoid increasing training difficulty at the same time, we adopt the widely used residual mechanism in the residual networks [25]. Through the shortcut connection, the residual mechanism reformulate the neural network as learning the residual functions according to the inputs. It has been proved that the residual structure is easier to be optimized, and can obtain the accuracy from the increased neural network’s depth [25]. The feature enhancing process is shown in Equation (7), where $X_{out}$ denotes the output of the proposed feature enhancing module.

(7) $$X_{out}=X+Y_{W}$$

In this section, on the basis of the feature enhancing process and the residual mechanism, we emphasize the important features and avoid increasing training difficulties at the same time. In addition, the feature enhancing module maintains the dimensions of the inputs and the outputs of the original feature maps. The dimension maintaining ability makes the proposed feature enhancing module a more generic extension, which can be added to the original neural network.

## 5. Simulations

### 5.1. Settings of the Radio Environment

In the practical wireless radio environment, the large scale fading includes two factors: the radio propagation loss and the shadow fading. In the simulations, we adopt the inverse polynomial law model $\gamma_{pr}=\min\left\{1,{\left(d/d_{c}\right)}^{-\alpha }\right\}$ as the radio propagation loss model [27]. $\gamma_{pr}$ is the propagation loss from the transmitting PU to the receiving SU. *d* is the distance between PU and SU. The preselected constants, $d_{c}$ and α, depend on the wireless radio environment. In addition, we use the log normal distribution model with zero mean and $\sigma_{sd}^{2}$ variance to simulate the shadow fading effect in the target region.

**Figure 8:**
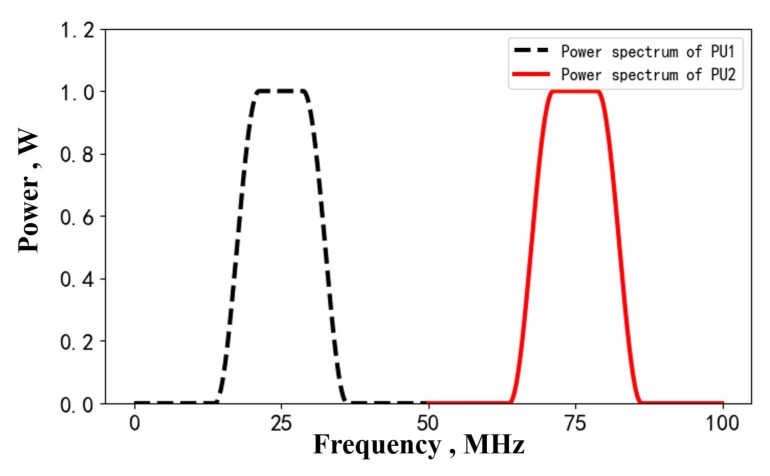
The power spectrum of PU1 and PU2 in the testing set.

We divide the testing area ***T*** into 48×48 grids. Regarding the testing data set, we assume α=2, $d_{c}\;=\;2$ and $\sigma_{sd}^{2}=1$ for the target area ***T***. We assume that two transmitting primary users are located at grids (20,18) and (40,35), under the AWGN with known variance ${\sigma_{0}}^{2}$. The receiving secondary users are uniformly distributed in ***T***. The number of SUs is about 15% of all 48×48 grids. The PUs are transmitting random signals. By sampling the PUs’ signals, their power spectrum can be obtained on the basis of the periodogram algorithm. In our simulation settings, the power spectrums of PU1 and PU2 are directly set, as shown in Figure 8, which center at 25 MHz and 75 MHz.

As for the training data set, we use the same radio environment model but different parameters. We generate 20,000 training images from two sets of propagation parameters: (1) α=2, $d_{c}\;=\;1$ and $\sigma_{sd}^{2}=1$; (2) α=1, $d_{c}\;=\;2$ and $\sigma_{sd}^{2}=0.5$. Each set includes 10,000 images under the AWGN with known variance ${\sigma_{0}}^{2}$. The number of active PUs in each radio environment map is randomly selected from 1 to 5 independently. The transmitting power of PU is normalized to 1 W. We suppose that the secondary users are uniformly distributed and adopt the random pixels sampler with $\alpha_{0}=0.15$. In addition, we avoid overfitting in a data augmentation way. During the training process, the images are sequentially and randomly transformed through 3 operations: horizontally flipping, vertically flipping and image transposing. The above 3 operations will make the training set 8 times larger than the original data set.

### 5.2. Settings of the PRF Algorithm

In the PRF training process, the learning parameters are as follows: we use Adam algorithm and the learning rates of *D* and *G* are 0.0004 and 0.0001; the batch size is 24; the gradient penalty coefficient is 10 [23].

To monitor the estimation ability of the generator in the training process, we define the Euclid distance $d_{E}$ between $C^{e}$ and $C^{r}$ in Equation (8). $M_{r}^{i}$ and $M_{e}^{i}$ are the *i*th real and estimated REMs in every training batch. bs is the batch size.

(8) $$\begin{array}{c} d_{E}=\frac{1}{bs}\overset{bs}{\underset{i=1}{\sum }}{∥M_{r}^{i}-M_{e}^{i}∥}_{2} \end{array}$$

**Figure 9:**
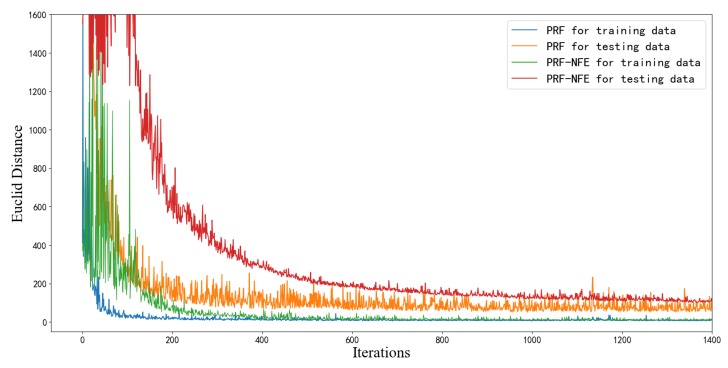
The convergence curves of PRF and PRF-NFE.

We use PRF-NFE to denote the PRF without the feature enhancing modules. Figure 9 shows the convergence curves of the PRF and the PRF-NFE algorithm. Each iteration includes 40 batches of the training images. The orange line denotes the estimation performance of the PRF algorithm for the testing data, which shows that we avoid the overfitting problem during the training process because of the data augmentation method.

As shown in Figure 9, the convergence performance of PRF (blue line) is better than that of PRF-NFE (green line) under the same number of iterations. The above convergence results are caused by the fact that the feature enhancing module can intelligently emphasize the important features and extract more information from the incomplete training data. The PRF algorithm has a stronger feature extracting ability than the PRF-NFE algorithm because of the feature enhancing module, which promotes the convergence during the training process.

In addition, although PRF converges faster than PRF-NFE from the perspective of the number of iterations, the PRF needs more time than PRF-NFE per iteration. On the basis of the Intel Core i7-8750H processor and RTX 2060 graphics card, the PRF and PRF-NFE need 6.7 s and 2.5 s per iteration, respectively. We prefer the PRF algorithm for 3 reasons: (1) the PRF algorithm has a better estimation performance than PRF-NFE, as verified by the following subsection; (2) The training data are limited in most cases. As for the REMs estimation task, a neural network with stronger information extracting ability is important because of the limited and incomplete training images; (3) A computer with a more powerful calculation ability can solve the time-consuming problem for the training process of the PRF algorithm.

We compare the proposed PRF with the PRF-NFE, IDW and Kriging algorithm on testing data. We use the the Kriging with the exponential semi-variogram. As for the IDW algorithm, the power value of the inverse distance is set to be $p_{id}=3$.

### 5.3. Tests for the PRF Algorithm

We select three indicators to test the pixel regression framework: (1) The visual display of the radio environment maps; (2) the estimated power spectrum of primary users; (3) the average REMs estimating error (AREE) against different numbers of secondary users.

#### 5.3.1. The Visual Display of the Radio Environment Maps

**Figure 10:**
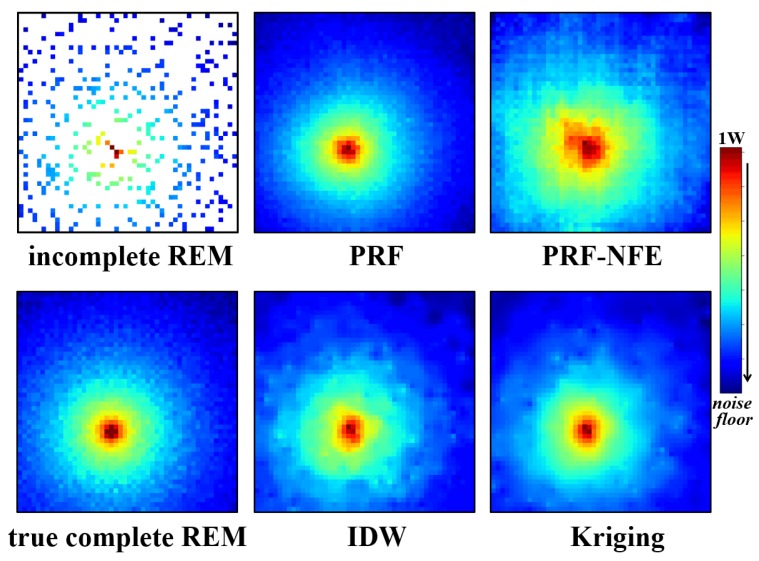
The incomplete radio environment maps, PRF, PRF without the feature enhancing module, IDW, Kriging with the exponential semi-variogram estimation results, and the true, complete radio environment map for PU1 at 25 MHz.

**Figure 11:**
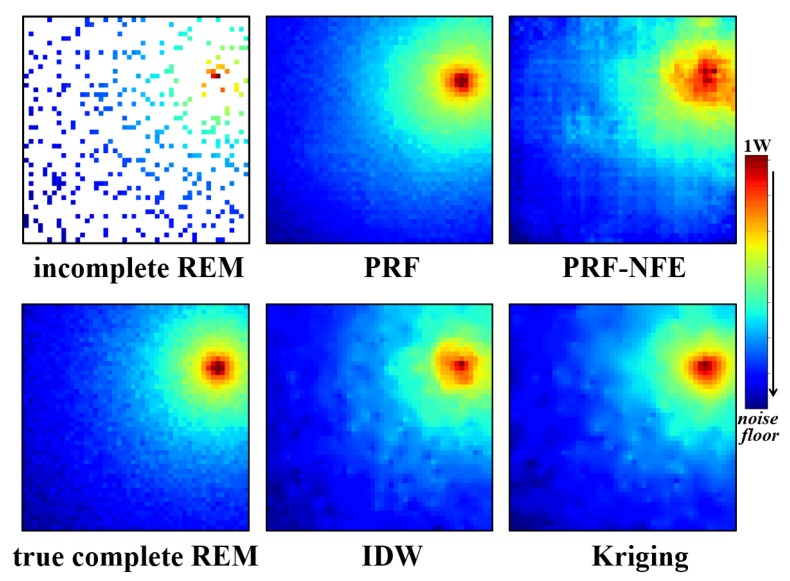
The incomplete radio environment maps, PRF, PRF without the feature enhancing module, IDW, Kriging with the exponential semi-variogram estimation results and the true, complete radio environment map for PU2 at 75 MHz.

The test for the visual display of the radio environment maps is relatively simple. We directly input the incomplete REMs in the target area to the well-trained generator. Then we observe the estimation results from the generator. It is an intuitive testing method. The reconstruction performances for PU1 and PU2 are displayed in Figure 10 and Figure 11.

Compared with the true, complete radio environment maps, the PRF method achieves better estimation results than the IDW and Kriging method from the direct visual display, especially the estimated area near the source of radiations.

In addition, the PRF also outperforms the PRF without feature enhancing module (PRF-NFE), which demonstrates that the proposed module enhances the approximation ability and the feature extracting ability of the pixel regression framework.

#### 5.3.2. The Estimated Power Spectrum of Primary Users

**Figure 12:**
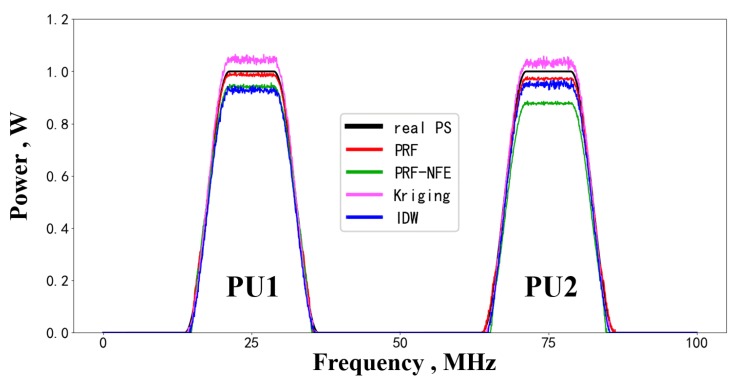
The power spectrum reconstruction results for PUs.

Regarding the performance of the estimated power spectrum, we compare the PRF reconstruction results with the true primary users’ power spectrum in Figure 12. The testing performance demonstrates the estimation ability of the PRF for the unused bands. Figure 12 shows that the PRF has a better reconstruction performance than PRF-NFE, Kriging and IDW interpolation.

The proposed PRF algorithm outperforms the PRF-NFE because of the feature enhancing module, which enhances the approximation ability and the feature extracting ability during the same training epochs.

Regarding the Kriging interpolation algorithm in Figure 12, the deviation comes from the biased spatial features hypotheses upon the radio environment (i.e., the semi-variogram function assumptions) about the target area. However, spatial features hypotheses upon the wireless environment are core factors for the radio environment maps reconstruction.

As for the IDW algorithm, the influences of the available nodes on the estimated nodes are controlled by the power value of the inverse distance, i.e., ${\left(\frac{1}{d_{idw}}\right)}^{p_{id}}$. Inaccurate setting of $p_{id}$ will lead to imprecise radio environment maps. In fact, it is difficult to decide whether a certain $p_{id}$ is appropriate or not.

#### 5.3.3. The Average REMs Estimating Error Against Different Numbers of Secondary Users

As for the average REMs estimating error (AREE) against different numbers of the sensing nodes, we choose $a_{f}$ frequency points randomly within the PUs’ working spectrum. Then we define the AREE in Equation (9).

(9) $$\begin{array}{c} AREE=\frac{1}{a_{f}}\overset{a_{f}}{\underset{f=1}{\sum }}{∥M_{true}^{f}-M_{estm}^{f}∥}_{2} \end{array}$$

where $M_{true}^{f}$ and $M_{estm}^{f}$ are the true complete radio environment maps and the estimated results at the *f* th frequency point. The radio environment maps are reconstructed on the basis of the power spectrum from the receiving users. The more power spectrum from receiving users, the better estimated performance from the pixel regression framework. Thus, the AREE is related to the numbers of the sensing nodes.

**Figure 13:**
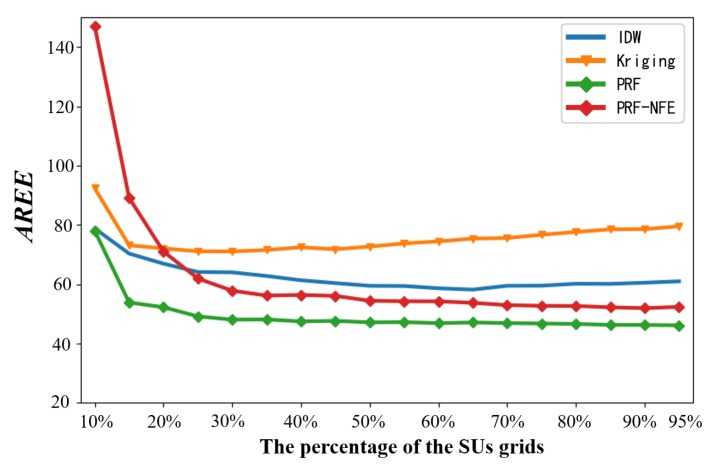
The average REMs estimating error against different numbers of secondary users.

We choose the estimated REMs randomly at 20 frequency points and calculate AREE against different numbers of secondary users. Figure 13 shows the simulation results.

The average REMs estimating errors of the PRF and the PRF-NFE decrease with the increase of the number of secondary users gradually in Figure 13. The above testing results demonstrate that the more PS from the sensing nodes, the better the reconstruction performance from the PRF algorithm. In the range of 10% to 95%, the PRF algorithm has a better estimation results than the PRF-NFE, IDW and Kriging algorithm.

Compared with the PRF-NFE, the PRF performs better because of the feature enhancing module, which helps the neural network to extract more information from the incomplete training data. In addition, the reconstruction performance of PRF is not good enough at 10%. It is mainly caused by the fact that the amounts of the PS measurements are too small to activate the neural network of the PRF in the data forward propagation process. To solve this problem, we can select a smaller value to the parameters of the measurement function in the PRF training process, e.g., $\alpha_{0}=0.05$.

Regarding the IDW method, it performs worse compared with PRF. The inaccurate reconstruction result is from the imprecise power value setting in IDW, which controls the influence of the receiving users on the interpolation points.

As for the AREE of the Kriging algorithm, it increases after an initial decrease because: (1) The PS measurements contain a little amount of information in the beginning for Kriging. The AREE decreases from 10% to 25% because of the increasing information of the PS measurements from secondary uses. (2) During the increase from 25% to 95%, the AREE increases because the more PS measurements from secondary uses, the larger deviations between the Kriging semi-variogram and the true complex wireless environment.

## 6. Conclusions

In this paper, we propose a generative adversarial networks-based radio environment maps estimation algorithm named pixel regression framework. The proposed PRF algorithm relaxes the requirement of the complete training images in the traditional deep learning-based method and can estimate the REMs using only incomplete REMs training data. To improve the feature extraction ability for the incomplete REMs, we design a feature enhancing module for the PRF algorithm, which intelligently learns and emphasizes the important features from the REMs images. We simulate a radio environment with the radio propagation loss and the shadow fading, and select three indicators to test the proposed algorithm: the visual display of the radio environment maps, the estimated power spectrum of PUs, and the average REMs estimating error against different numbers of secondary users. Compared with the traditional methods, the PRF algorithm performs better upon the above indicators in the simulations. In our future research, we will concentrate on utilizing the correlation information between adjacent frequencies’ training images to improve the REMs estimation performance.

## Figures and Tables

**Table 1:** The list of acronyms.

Abbreviation	Full Name
REMs	radio environment maps
PS	power spectrum
GAN	generative adversarial network
PRF	pixel regression framework
CR	cognitive radio
CRN	cognitive radio network
PU	primary user
SU	secondary user
AWGN	additive white Gaussian noise
IDW	inverse distance weighted
DNN	deep neural network
FE	feature extracting
FW	feature weighting
G	generator
D	discriminator
WGAN-GP	Wasserstein GAN with gradient penalty
Adam	adaptive moment
